# Renal Transplantation Could Reverse Dialysis-Associated Porphyria

**DOI:** 10.7759/cureus.28482

**Published:** 2022-08-27

**Authors:** Sasanka k Barua, Sachinkumar Patel, Debanga Sarma, Mandeep Phukan, Puskal k Bagchi

**Affiliations:** 1 Urology, Gauhati Medical College & Hospital, Guwahati, IND

**Keywords:** porphyrin profile, hemodialysis, renal transplantation, n-acetylcysteine, porphyria cutanea tarda, pseudoporphyria

## Abstract

Pseudoporphyria (PP) is a relatively infrequent, photodistributed bullous dermatosis that clinically, histopathologically, and immunologically resembles porphyria cutanea tarda (PCT), but is not accompanied by porphyrin abnormalities in the serum, urine, or stool. It was initially described in patients with renal failure on dialysis as 'bullous dermatosis of hemodialysis.' Pseudoporphyria has been seen in patients with end-stage renal disease on hemodialysis. No treatment has proved efficacious in the management of pseudoporphyria. However, N-acetylcysteine has been anecdotally reported to be effective in the management of hemodialysis-related pseudoporphyria and other porphyric diseases. Our patient had developed multiple skin lesions all over the body when hemodialysis started. The lesions were erythematous with fluid-filled vesicles, and bullae with cutaneous fragility that were evaluated and diagnosed as pseudoporphyria. The patient was treated with available all medication in the literature but was not relieved. However, all skin lesions completely healed within 22 days post renal transplantation. Renal transplantation proved to be the cure for dialysis-induced pseudoporphyria resistant to conventional drug therapy.

## Introduction

Porphyrias are heme synthesis metabolic diseases. Excessive buildup and excretion of 5-aminolevulinic acid, porphobilinogen, and/or porphyrins arise from partial enzymic deficits. Porphyria is a collective term for a collection of eight heme biosynthesis pathway metabolic diseases [1,2]. In North America and Europe, porphyria cutanea tarda (PCT) is the most frequent porphyria. This blistering illness is caused by a lack of uroporphyrinogen decarboxylase, a heme biosynthetic enzyme, that was first reported by Waldenström in 1937 [3]. Pseudoporphyria (PP) is a photodistributed bullous dermatosis that resembles PCT clinically, histopathologically, and immunologically, but is not accompanied by porphyrin abnormalities in the serum, urine, or stool. It was first reported as 'bullous dermatosis of hemodialysis' in patients with renal insufficiency who were on dialysis [4]. Pseudoprphyria is thought to affect 1.2% to 18% of patients on hemodialysis and a smaller percentage of those on peritoneal dialysis with end-stage renal failure. Pseudoporphyria has been linked to a variety of factors, the most common of which include ultraviolet light exposure, nonsteroidal anti-inflammatory medications, diuretics, and chronic renal failure in patients receiving or not receiving dialysis [4]. Although N-acetylcysteine has been anecdotally reported to be beneficial in the management of hemodialysis-related pseudoporphyria and other porphyric diseases [5,6,7], no medication has been effective in its management. We describe a case of N-acetylcysteine resistance caused by hemodialysis that was effectively treated by kidney transplantation. Chloroquine has also been tried and found to be effective [8].

## Case presentation

A 30-year-old woman was taken to the nephrology department after experiencing edema in both her lower limb and face for 10 months. She was assessed and diagnosed with chronic kidney disease (grade V) due to hypertension. She also had dilated cardiomyopathy and a low ejection fraction of 28%. She had been on traditional hemodialysis for eight months. Hemodialysis was performed on the patient about 50 times in total and was scheduled three times a week for four hours each time, using an arteriovenous fistula in the left forearm.

**Figure 1:**
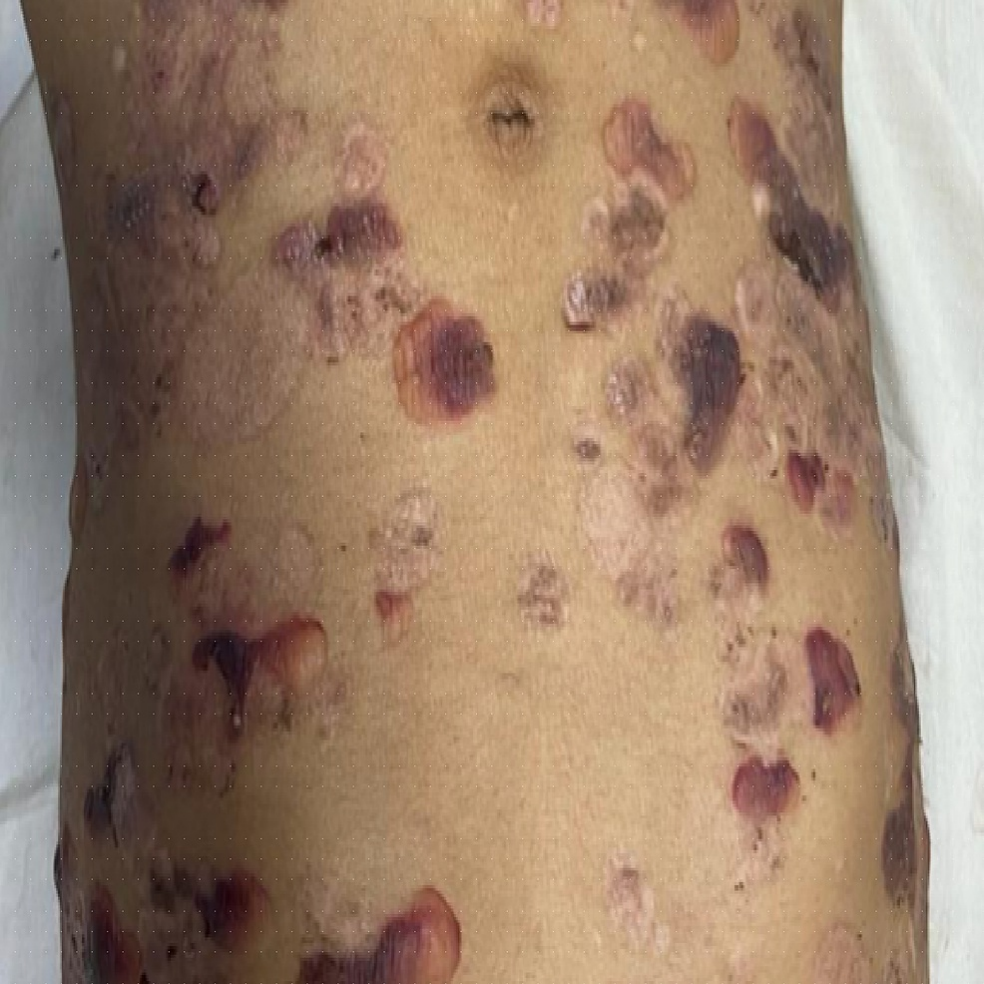
Pseudoporphyric lesions over the skin of the anterior abdominal wall

**Figure 2:**
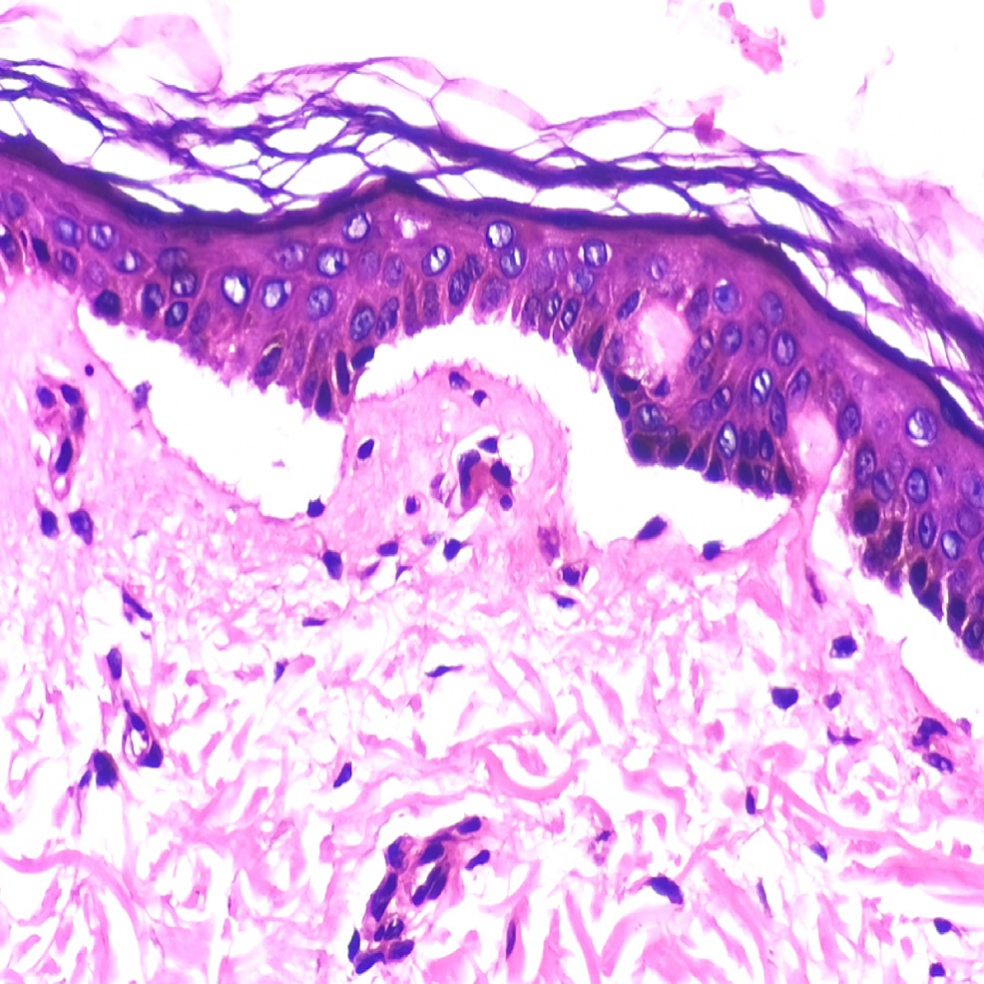
Histopathological features of pseudoporphyria

Following the hemodialysis, the patient acquired erythematous skin lesions all over the body with fluid-filled vesicles and bullae with cutaneous fragility (Figure 1), for which dermatological consultation was sought and assessed. After a thorough examination and ruling out other potential causes of porphyria (the porphyrin profile was within normal limits), a biopsy of the lesion revealed inflammatory poor sub-epidermal vesicular dermatitis. Further immunofluorescence revealed a linear deposit of C3 at the dermo-epidermal interface, as well as the absence of immunoglobulin (IG) A, IGG, IGM, and C1Q (Figure 2). The serum antinuclear antibody (ANA) profile was within normal ranges in blood testing. Clinical and histological evidence led to the diagnosis of pseudoporphyria. The woman was given topical corticosteroids and 600 mg of N-acetylcysteine orally daily for a month, but no improvement was seen in her skin condition, and new lesions continued to emerge all over her body.

**Figure 3:**
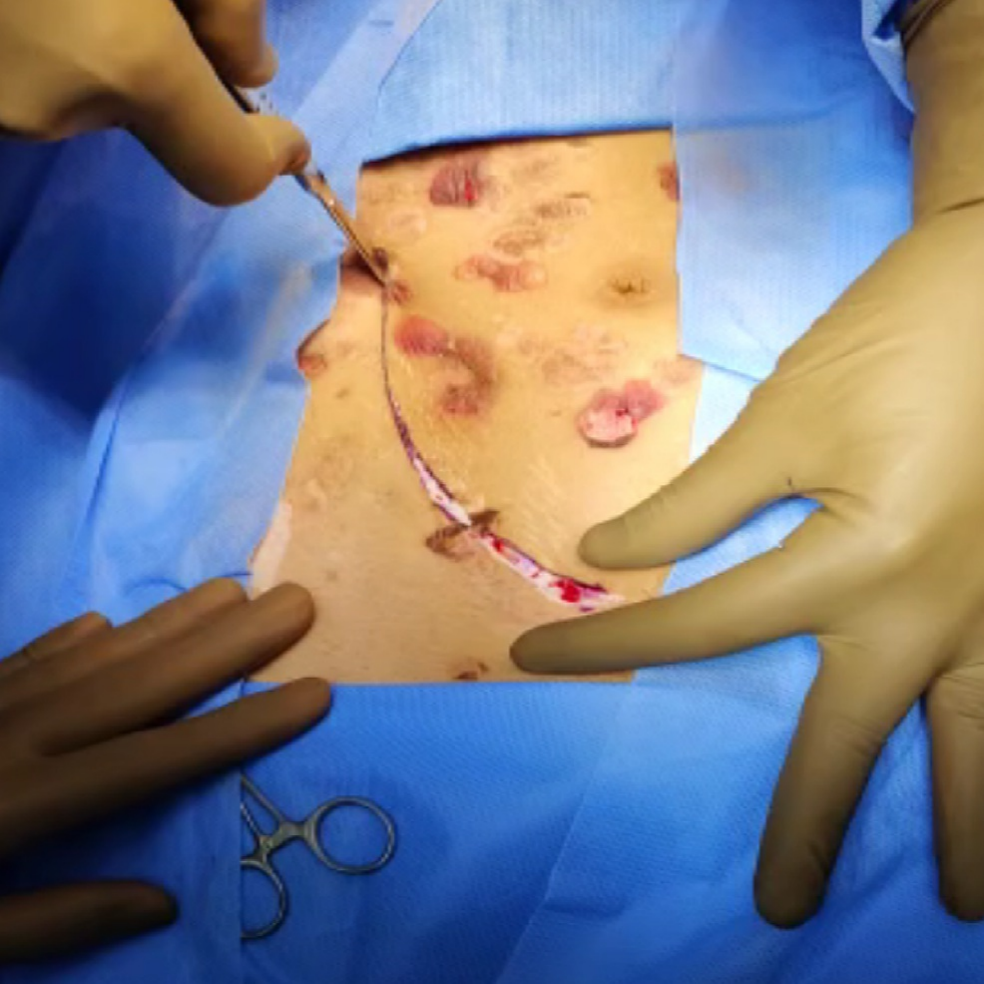
Incision made over the skin with pseudoporphric lesion for kidney transplant

**Figure 4:**
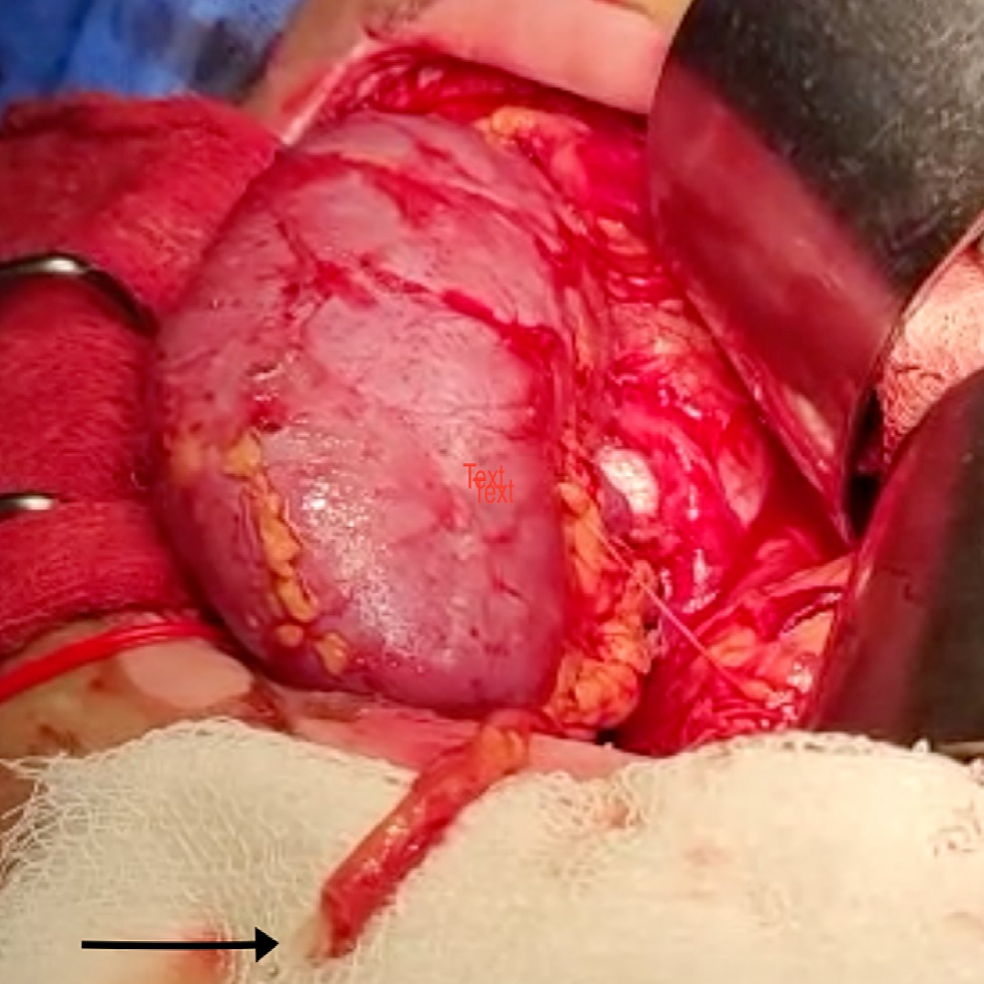
Black arrow points to urine pouring out from the ureter intraoperatively after declamping the vessels post anastomosis completion

After prepping her skin with a diluted iodine solution (Figure 3), the patient had a live donor renal transplantation. The external iliac artery was anastomosed with the renal artery. A renal vein was anastomosed with the external iliac vein end-to-side with prolene 6-0 double-arm, and ureteric implantation was performed across the fundus of the urinary bladder using the modified Lich Gregor technique. The transplanted kidney's warm ischemia time was eight minutes, and the cold ischemia period was 46 minutes. Within a minute of declamping vessels, the kidney began to flow urine intraoperatively (Figure 4). On the first post-operative day, an ultrasonogram (USG) Doppler scan of the transplanted kidney revealed acceptable vascularity.

The patient's serum creatinine level was 5.4 mg/dl before surgery. After kidney transplantation, serum creatinine declined to 2.2 mg/dl on postoperative day (POD) one, to 1.22 mg/dl on POD two, and subsequently decreased to a normal value of 0.55 mg/dl. On POD one, the patient's urine production was 7190 mL per 24 hours, 6400 mL per 24 hours on POD two, 7330 mL per 24 hours on POD three, and subsequently decreased to 3840 mL per 24 hours on POD 12. The patient's drain output averaged 100 mL to 150 mL per 24 hours before decreasing to 20 mL per 24 hours on POD 3. On POD 14, the per-urethral catheter was withdrawn.

**Figure 5:**
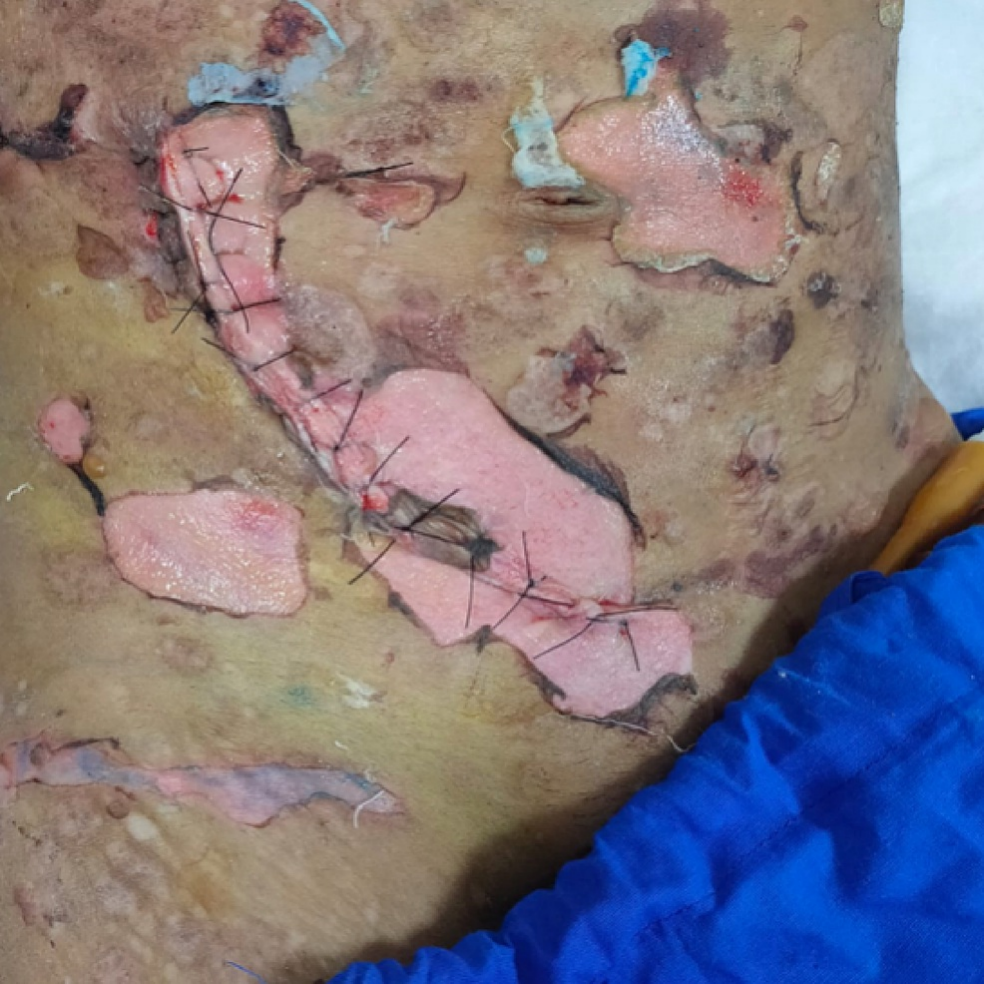
The operated site and skin lesions on postoperative day five

**Figure 6:**
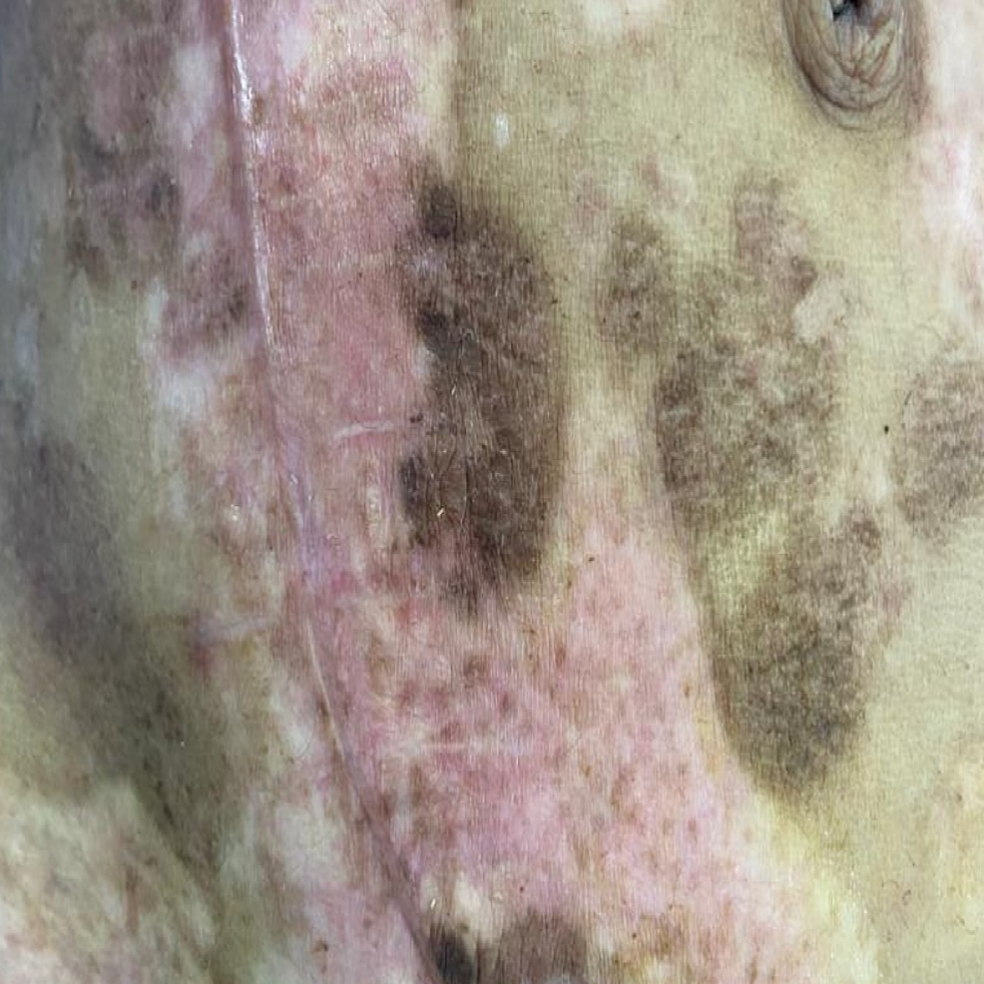
Healed skin lesions and operated site on postoperative day 22

From POD three onwards, the patient's skin lesions began shrinking and healing (Figure 5), and no new lesions were discovered anywhere on her body. On POD five, 30% of skin lesions began to regress, and on POD seven, roughly 50% of skin lesions began to resolve. By POD 22, practically all skin lesions had disappeared (Figure 6), and no new lesions had appeared throughout the body. The operation site, too, healed properly with no signs of infection.

## Discussion

In pseudoporphyria, bullae form on photo-exposed skin, most commonly on the dorsum of the hands and feet, forearms, face, and neck. As the lesions heal, scarring and milia will appear. The diagnosis of pseudoporphyria requires the elimination of true porphyria, notably PCT. By definition, the porphyrin profile in PP is normal or near normal [9]. This was noticed in our case. Serum porphyrin levels in chronic renal failure patients are higher than in the general population, with some values falling within the lower end of the range reported in PCT patients with normal renal function. Furthermore, plasma uroporphyrin levels are frequently higher in hemodialysis patients than in peritoneal dialysis patients, which could explain why the latter group had a lower incidence of PP.

Pseudoporphyria's exact pathophysiological mechanism is still a mystery. Renal failure, ultraviolet A radiation, and photosensitizing drugs (naproxen, furosemide, nalidixic acid, bumetanide, tetracyclines, amiodarone, and others) have all been related to pseudoporphyria [4]. When numerous contributory factors such as regularly used photosensitizing medicines are present in hemodialysis patients, the risk of pseudoporphyria may be increased [10]. The mainstay of treatment is the discontinuation of the suspected photosensitizing drug and stringent ultraviolet A protection. Previous studies have shown that N-acetylcysteine and high-flux hemodialysis can effectively treat pseudoporphyria [11]. Our patient, however, did not respond to either therapy, and new skin lesions emerged often. After kidney transplantation, no new skin lesions appeared and those already present shrunk in size as they resolved, with almost all lesions healing within 22 days postoperatively.

The question of whether the disease (chronic renal failure) or the treatment (hemodialysis) plays a bigger role in pseudoporphyria still needs to be answered. However, in our patient, lesions did not show any indication of clearing even post N-acetylcysteine therapy, most likely due to hemodialysis-induced skin lesions. Such cases support the theory that renal impairment is a more essential aetiological factor in the development of pseudoporphyria than previously considered. Renal transplantation, on the other hand, continued to improve our patient's quality of life by enhancing the near-total healing of skin lesions as well as normalising her renal function. It's likely the first case of pseudoporphyria caused by dialysis that has been successfully treated with kidney transplantation.

## Conclusions

Pseudoporphyria has many causes, the most prevalent being UV exposure, non-steroidal anti-inflammatory drugs (NSAIDs), diuretics, and chronic renal failure with or without hemodialysis. The use of N-acetylcysteine to treat PP showed no evidence of resolving the lesions on our patient. Renal transplantation proved to be the cure for our patient's dialysis-induced pseudoporphyria that was resistant to conventional drug therapy with N-acetylcysteine, and the transplantation contributed to a near total resolution of the dermal lesions within a short span of time. Therefore, it has been proven through our case report that by renal transplantation both pseudoporphyria and chronic kidney disease (CKD) can be treated as early as possible and improve the quality of life of patients.
